# Ribosomal S6 kinases determine intrinsic axonal regeneration capacity

**DOI:** 10.1371/journal.pbio.3002094

**Published:** 2023-04-21

**Authors:** Wilfredo Mellado, Dianna E. Willis

**Affiliations:** 1 Burke Neurological Institute, White Plains, New York, United States of America; 2 Feil Family Brain & Mind Research Institute, Weill Cornell Medicine, New York, New York, United States of America

## Abstract

Why do adult mammalian central nervous system axons not regenerate? This Primer explores two recent PLOS Biology manuscripts that have revealed a role for two related ribosomal S6 kinases, RSK1 and RSK2, in the regenerative capacity of the mammalian central and peripheral nervous systems.

## Teaser

Why do adult mammalian central nervous system axons not regenerate, when peripheral axons do? Two studies in *PLOS Biology* point to the role of 2 related ribosomal S6 kinase family members in the differences in regeneration capacity between central and peripheral axons.

It has long been appreciated that mature neurons of the adult mammalian central nervous system (CNS) differ from those of invertebrates such as *Caenorhabditis elegans* and lower vertebrates such as fish, and from adult mammalian peripheral nervous system (PNS) neurons in their capacity to regenerate axons following injury [1–3]. Axonal regeneration in the CNS is limited by a number of factors. These have broadly been classified into extrinsic and intrinsic factors. Extrinsic factors, such as inhibitory factors present within the CNS, a lack of growth-promoting factors within the CNS environment, and the scar formation and inflammatory response triggered following injury, have all been hypothesized either individually or in combination to underlie the failure of CNS axonal regrowth [4]. While overcoming these extrinsic factors has been the focus of considerable research over the past 2 decades, it has become clear that neurons in the adult mammalian CNS have limited intrinsic regenerative capacity, and it is this inherent reduced injury response that may prevent CNS axonal regeneration [5].

Failure of injured CNS axons to regenerate is likely due to the complex interplay between both extrinsic and intrinsic factors. In the past decade, several intrinsic regulators of CNS axon regeneration have been identified. The most promising of these has been the mammalian target of rapamycin (mTOR), a conserved serine/threonine kinase that has a crucial role in regulating various cellular processes, including cell growth, proliferation, and survival. mTOR regulates CNS axonal regeneration by integrating multiple signaling pathways to promote axon growth and regeneration, including by promoting protein synthesis by activating p70 ribosomal S6 kinase (S6K) and eukaryotic initiation factor 4E-binding protein (4E-BP), which leads to increased synthesis of growth-promoting proteins required for axon regeneration [6]. While this pathway has shown promise in regulating intrinsic regenerative capacity of CNS neurons, 2 recent studies in *PLOS Biology* highlight the importance of the p90 ribosomal S6 kinase (RSK) family members in controlling axonal regeneration and may point to an important mechanism driving the dichotomy between the regenerative responses in the PNS and CNS [7,8].

RSKs are a family of serine/threonine protein kinases that catalyze the phosphorylation of target proteins on specific serine or threonine residues. There are 4 isoforms (RSK1-4), each encoded by separate genes. RSKs themselves are activated by phosphorylation, and upon activation, they subsequently phosphorylate a variety of downstream targets, including ribosomal protein S6 (RPS6), nuclear transcription factors, and cytoskeletal proteins. In addition to their role in normal cellular processes including cell growth, proliferation, differentiation, survival, and motility, RSKs have been implicated in several diseases, such as cancer, diabetes, and neurological disorders [9]. A recent paper by Mao and colleagues found that one of the isoforms, RSK1, is up-regulated in PNS neurons following axonal injury [7]. Inhibition of RSK1 reduced axonal regeneration while overexpression enhanced growth, pointing to both the necessity and sufficiency of RSK1 for PNS axonal regeneration. When RSK1 levels were inhibited, the growth attenuation could be rescued by overexpressing the elongation factor eEF2, leading to increased translation of pro-regenerative proteins. The authors hypothesize that axonal injury up-regulates PNS neuronal levels of RSK1, which phosphorylates eEF2 kinase and results in activation of eEF2 to increase translation of pro-regenerative proteins. Interestingly, inhibition of mTOR in these peripheral neurons had no effect on axonal growth, suggesting that RSK1-regulated increase in pro-regenerative protein synthesis can be mTOR independent. In CNS neurons, axonal injury did not result in an increase in the levels or activity of RSK1. This may explain, in part, the difference in intrinsic regenerative capacity between PNS and CNS neurons. Perhaps surprisingly, overexpression of RSK1 alone in retinal ganglion cells was not able to promote regeneration of CNS axons following optic nerve injury, highlighting the complexity of the differences between PNS and CNS intrinsic regenerative capacity.

The data shown by Mao and colleagues [7] demonstrates the key role that the regulation of neuronal protein synthesis has in driving axonal regeneration. A new paper in this issue of *PLOS Biology* further strengthens the role of regulation of protein synthesis in general, and the importance of the RSK family in particular, in this process. In this paper, Decourt and colleagues demonstrate that axonal injury in the PNS leads to phosphorylation of RPS6 [8]. Phosphorylation of RPS6 at position 235/236 is a well-known downstream target of mTOR and a marker of mTOR pathway activation, which results in increased protein synthesis, cell growth, and proliferation. RPS6 phosphorylation is known to correlate with regenerative capacity in both the PNS and the CNS, and its levels are known to decrease during development and with age, concomitant with the decrease in regenerative capacity [10]. Understanding the mechanism(s) by which the levels of phosphorylated RPS6 are regulated becomes an attractive target for understanding what drives intrinsic regenerative capacity.

Similar to Mao and colleagues, Decourt and colleagues [8] show that phosphorylation of RPS6 at position 235/236 is not controlled by mTOR but rather by RSK2, another RSK family member. In addition to its role in driving PNS axonal regeneration, they further demonstrate that RSK2 can promote CNS regeneration in dorsal column neurons. This is in contrast to the results of Mao and colleagues [7] showing that RSK1 overexpression alone could not enhance CNS axonal growth in retinal ganglion neurons. So, while both studies demonstrate that RSK1 and RSK2 are important in PNS regeneration, their contributions to CNS regeneration are divergent. While RSK1 and RSK2 share many of the same functions and signaling pathways, their specific downstream targets are not completely shared. Furthermore, the downstream targets of RSK1 and RSK2 may also differ between species and in different cell types. In lower organisms, they primarily regulate transcription factors and other signaling proteins that are involved in cell growth and development. In contrast, in higher mammals, they have been shown to regulate a broader range of targets, including cytoskeletal proteins, ion channels, and other proteins involved in neuronal function. While both studies used rodent species, there may also be differences in the role of RSK1 and RSK2 in rats (as used by Mao and colleagues) and mice (as used by Decourt and colleagues), or in the different CNS neuronal cell types studied. Other examples of divergent CNS regeneration programs in different neuron types within the same species have been demonstrated [1], underscoring the complexity of axonal regeneration.

**Fig 1:**
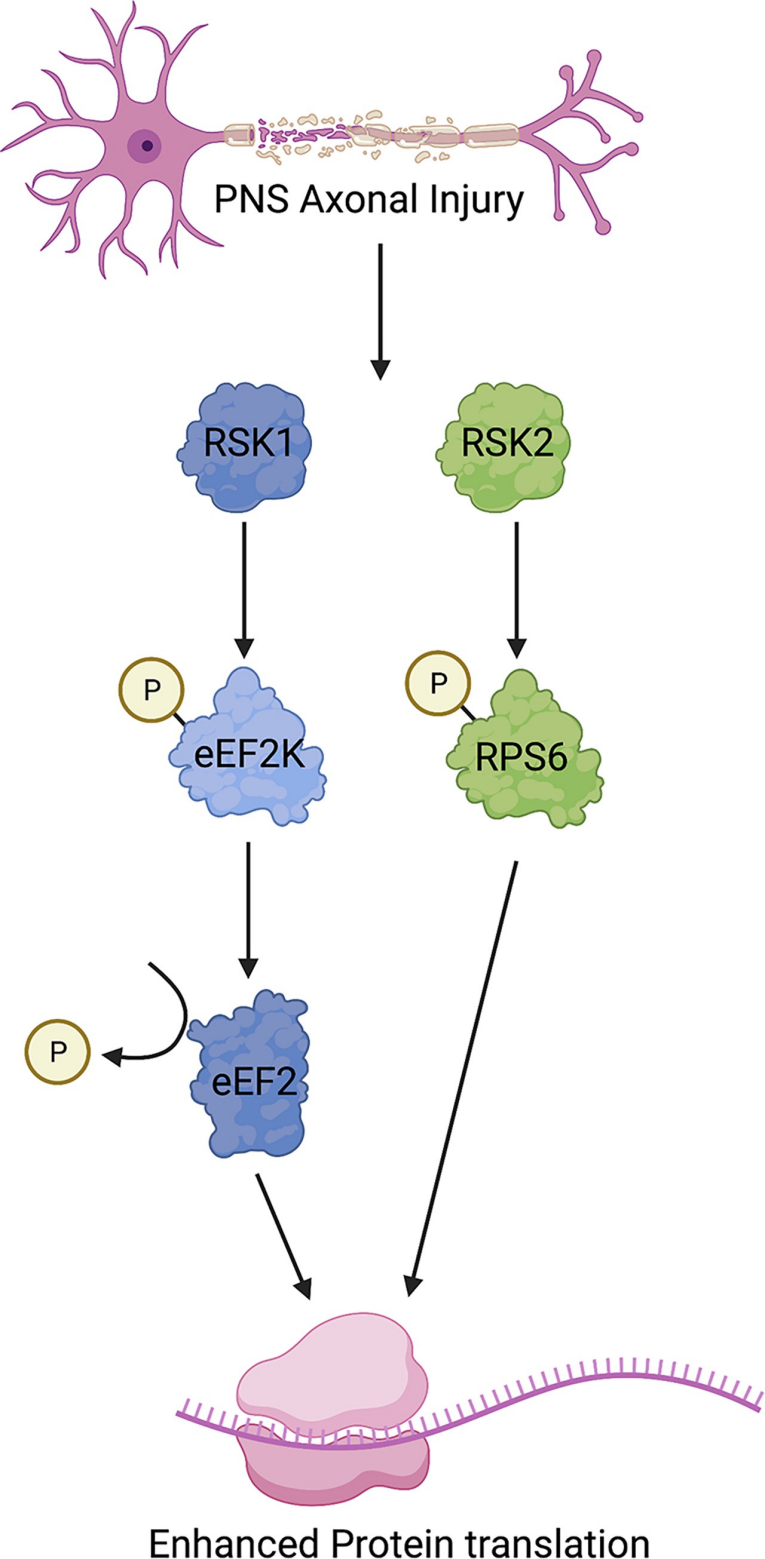
RSK family members drive enhanced protein synthesis in PNS neurons following axonal damage. In contrast to the CNS, axonal injury in the PNS leads to an up-regulation and activation of RSK family members RSK1 and RSK2. Activation of RSK1 leads to phosphorylation of the known mTOR target eEF2K, allowing for the dephosphorylation and derepression of eEF2. This results in increased translation of pro-regenerative proteins such as BDNF. Similarly, activation of RSK2 leads to phosphorylation of the know mTOR target RPS6 at positions 235/236, again driving increased translation. Both of these activation pathways are independent of mTOR. It remains to be determined how these 2 pathways may synergize to drive the intrinsic growth capacity of PNS neurons. Since activation of RSK1 alone in retinal ganglion CNS neurons is not sufficient to drive axonal growth while activation of RSK2 is sufficient promote CNS regeneration in dorsal column neurons the extent to which RSK family members may be able to enhance CNS regeneration is still unclear. Created with BioRender.com. BDNF, Brain Derived Neurotrophic Factor; CNS, central nervous system; eFEF2, eukaryotic elongation factor 2; mTOR, mammalian target of rapamycin; PNS, peripheral nervous system; RPS6, ribosomal protein S6; RSK, p90 ribosomal S6 kinase.

While the exact contribution of RSK1 and RSK2 to PNS and CNS axonal regeneration in adult mammalian neurons remains to be fully elucidated, these 2 papers [7,8] highlight the importance of RSK-mediated regulation of translation in the intrinsic growth capacity of neurons (Fig 1). Interestingly, they may also point to a complexity in the response of different CNS neuron types that has not been previously fully appreciated. The heterogeneity of intrinsic capacity of different CNS neuron types even within a single species, such as those found in the retina compared to those in the spinal cord, has been well documented [1]. Considerable effort has been centered on understanding the key factors that may drive this intrinsic regenerative capacity variability. Taken together, the papers of Mao and colleagues and Decourt and colleagues enhance our understanding of the differences in intrinsic axonal growth capacity in adult mammalian PNS and CNS neurons and suggest that further research into the ability of different CNS neuronal types to engage the RSK1- or RSK2-mediated pathways may hold the key to unlocking enhanced CNS regeneration.

## References

[1] Fu XQ, Zhan WR, Tian WY, Cao DD, Luo ZG. Intrinsic heterogeneity in axon regeneration. Biochem Soc Trans. 2022;50(6):1753–1762. doi: 10.1042/BST20220624 36382964

[2] Rasmussen JP, Sagasti A. Learning to swim, again: Axon regeneration in fish. Exp Neurol. 2017;287(Pt 3):318–330. doi: 10.1016/j.expneurol.2016.02.022 26940084

[3] Zheng B, Tuszynski MH. Regulation of axonal regeneration after mammalian spinal cord injury. Nat Rev Mol Cell Biol. 2023. doi: 10.1038/s41580-022-00562-y 36604586

[4] Fawcett JW. The Struggle to Make CNS Axons Regenerate: Why Has It Been so Difficult? Neurochem Res. 2020;45(1):144–158. doi: 10.1007/s11064-019-02844-y 31388931 PMC6942574

[5] He Z, Jin Y. Intrinsic Control of Axon Regeneration. Neuron. 2016;90(3):437–451. doi: 10.1016/j.neuron.2016.04.022 27151637

[6] Zhang J, Yang D, Huang H, Sun Y, Hu Y. Coordination of Necessary and Permissive Signals by PTEN Inhibition for CNS Axon Regeneration. Front Neurosci. 2018;12:558. doi: 10.3389/fnins.2018.00558 30158848 PMC6104488

[7] Mao S, Chen Y, Feng W, Zhou S, Jiang C, Zhang J, et al. RSK1 promotes mammalian axon regeneration by inducing the synthesis of regeneration-related proteins. PLoS Biol. 2022;20(6):e3001653. doi: 10.1371/journal.pbio.3001653 35648763 PMC9159620

[8] Decourt C, Schaeffer J, Blot B, Paccard A, Excoffier B, Pende M, et al. The RSK2-RPS6 axis promotes axonal regeneration in the peripheral and central nervous systems. PLoS Biol. 2023; 21(*4*):e3002044. 10.1371/journal.pbio.3002044 37068088 PMC10109519

[9] Romeo Y, Zhang X, Roux PP. Regulation and function of the RSK family of protein kinases. Biochem J. 2012;441(2):553–569. doi: 10.1042/BJ20110289 22187936

[10] Biever A, Valjent E, Puighermanal E. Ribosomal Protein S6 Phosphorylation in the Nervous System: From Regulation to Function. Front Mol Neurosci. 2015;8:75. doi: 10.3389/fnmol.2015.00075 26733799 PMC4679984

